# Soluble TREM2 Mediates Blood Brain Barrier Permeability through Astrocyte Reactivity

**DOI:** 10.21203/rs.3.rs-10730833/v1

**Published:** 2026-08-27

**Authors:** Samuel Martinez-Meza, Brandon A. Sealy, Lillie Lopez, Douglas F. Nixon, Joan W. Berman

**Affiliations:** 1 Department of Pathology, Albert Einstein College of Medicine, Bronx, NY, USA; 2 Department of Microbiology and Immunology, Albert Einstein College of Medicine, Bronx, NY, USA; 3 Institute of Translational Research, Feinstein Institutes for Medical Research, Northwell Health, Manhasset, NY, USA.

**Keywords:** SolubleTREM2 (sTREM2), Astrocyte, Blood Brain Barrier (BBB), reactivation, permeabilization, Inflammation, tight junction (TJ), Matrix Metalloproteinases-2 (MMP2)

## Abstract

The Triggering receptor expressed on myeloid cells 2 (TREM2) is a transmembrane protein predominantly expressed by cells of the myeloid lineage. In the CNS, it is mainly expressed by microglia and macrophages, where it regulates their reactive state. This protein can be shed to release soluble TREM2 (sTREM2), which is increased in the cerebrospinal fluid of people with neurocognitive disorders. However, it remains unknown how sTREM2 contributes to neuropathogenesis. We examined the effects of sTREM2 on the inflammatory state of astrocytes, and how these impact blood–brain barrier (BBB) integrity using an in vitro BBB model composed of primary human Brain Microvascular Endothelial Cells (BMVEC) and primary human astrocyte cells. We found that sTREM2 induced an inflammatory state in astrocytes, characterized by increased AKT phosphorylation, NF-kB activation, and upregulation of activation markers including GFAP, all consistent with a reactive astrocyte (RA) phenotype. In addition, we found an increase in BBB permeability when astrocytes were exposed to sTREM2 that correlated with decreased levels of the tight junction (TJ) protein Occludin in BMVEC from the BBB. sTREM2 promoted release of astrocyte-derived molecules implicated in BBB disruption, with Matrix Metalloproteinases-2 (MMP2) appearing to be a major contributor to the loss of barrier integrity. Our findings demonstrate a novel role for sTREM2 in BBB dysfunction in the context of neuropathogenesis.

## Introduction

The triggering receptor expressed on myeloid cells 2 (TREM2) belongs to the immunoglobulin family on monocytes, macrophages, and microglia^1^. In microglia, TREM2 plays a key role in maintaining brain homeostasis by promoting the clearance of lipids, apoptotic cells, myelin debris, and other damage-associated molecules^2–4^. Additionally, it signals through the TYROBP/DAP12 adaptor to regulate microglial survival, metabolism, and activation state^5^. Proteolytic cleavage of TREM2 or alternative splicing generates a soluble form, soluble TREM2 (sTREM2), with proteolytic cleavage being the most common source^6^. Although less well-characterized than TREM2, sTREM2 was reported to promote neurocognitive disorders^7–9^, while other studies showed therapeutic benefits^10–13^. Higher levels of sTREM2 in cerebrospinal fluid have been associated with greater disruption of the blood–brain barrier (BBB), as measured by CSF/plasma albumin ratios, suggesting that sTREM2 may signal or participate in BBB damage and could serve as a marker of BBB disruption^9,14,15^. However, the mechanisms by which sTREM2 can disrupt the BBB remain minimally characterized.

In this study, we examined the impact of sTREM2 on the BBB by studying its effects on human astrocytes, a key component of the BBB. We showed that sTREM2 promotes a reactive state in human astrocytes, which correlated with a significant increase in permeability in the human BBB in vitro model, a model composed of human primary astrocytes and human primary BMVEC that has been extensively used to evaluate BBB in-vitro functions in several contexts^16–24^. This was characterized by decreased tight junction (TJ) Occludin in BMVEC from the same BBB model exposed to sTREM2. These findings provide the first evidence of mechanisms by which sTREM2 can contribute to BBB dysfunction and identifies the metalloproteinase MMP2 as a potential mediator of TJ degradation that might serve as a therapeutic target to limit BBB disruption.

## Methods

### Cell lines

Primary human cortical astrocytes were obtained a part of an approved research protocol at the Albert Einstein College of Medicine ^25^. These cells have been extensively characterized in terms of morphology and function in previous studies^26–31^. Human brain microvascular endothelial cells BMVEC were from cell-systems.

### Western Blotting

#### Western Blotting

Human astrocytes were lysed with RIPA buffer containing 1X Halt Protease Inhibitor and Phosphatase Inhibitor cocktail (Thermo Fisher Scientific). Lysate protein concentrations were quantified by Bradford Assay using Protein Assay Reagent Concentrate (Bio-Rad, Hercules, California). Equal amounts of protein from each condition for each donor were electrophoresed by SDS-PAGE under reducing conditions, with subsequent transfer to polyvinylidene difluoride (PVDF) membranes overnight at 4°C. Li-Cor Revert Total Protein Stain (Li-Cor, Lincoln, Nebraska) was used to quantify total protein optical density (O.D.), using the Li-Cor Odyssey Fc System for visualization, and Image Studio software (Li-Cor) version 5.2 for O.D. quantification. Membranes were blocked in SuperBlock Buffer (ThermoFisher) followed by overnight incubation at 4°C with mouse anti-phospho AKT (Cell Signaling Technology, Phospho-Akt (Ser473) (D9E) Rabbit Monoclonal Antibody #4060) and AKT (Cell Signaling Technology, Akt (pan) (C67E7) Rabbit Monoclonal Antibody #4691) in 5% BSA-TBST. HRP-conjugated anti-mouse IgG (Cell Signaling Technology, #7076) was used as secondary antibody for p-AKT, and HRP-conjugated anti-rabbit IgG (Cell Signaling Technology, #7074) was for AKT, each at 1:1000 dilution in 5% milk-TBST. Blots were developed using Super Signal West Femto Chemiluminescent Substrate (ThermoFisher) for phosphoproteins, or Western Lightning Plus ECL Oxidizing Reagent Plus (Perkin Elmer) for total AKT and STAT3 proteins.

### Western Blotting of BMVEC collected from the BBB

After 24 h of treatment, BMVEC protein expression was assessed by harvesting cells from 14 inserts per condition through gentle scraping of the upper surface of the transwell membrane. The pellet of cells was lysed and sonicated in RIPA buffer containing 1X Halt Protease Inhibitor and Phosphatase Inhibitor cocktail (Thermo Fisher Scientific). Lysate protein concentrations were quantified and all subsequent steps of western blott were carried out as described previously. After overnight incubation at 4°C with anti-rabbit Occludin, p-Claudin 5 or Total Claudin 5 (Cell Signaling) in 5% BSA-TBST, HRP-conjugated anti-rabbit IgG was used as secondary antibody in 5% milk-TBST. Blots were developed using Super Signal West Femto Chemiluminescent Substrate (ThermoFisher). Target proteins were normalized to total protein O.D. Data were analyzed as the fold change of normalized O.D. of target proteins in sTREM2 treated cells over the normalized O.D of target protein from cells treated with vehicle.

### The in vitro human BBB model

The in vitro blood–brain barrier (BBB) model has been extensively validated and used in many prior studies^16–24^. In this system, human primary BMVEC and human primary astrocytes are cocultured on opposite sides of a porous membrane insert (3 μm pores, Corning) in a 24-well plate. Through the membrane pores, astrocytic end feet establish contact with BMVEC, promoting formation of a tight barrier characterized by expression of TJ proteins and properties characteristic of the human BBB^32^. Prior to any treatment, multiple cocultures, established in parallel, were assessed for barrier permeability as quantified using Evans blue dye conjugated to albumin^33^.

### Permeability Assay

After the BBB-in vitro model was incubated with sTREM2 or control reagents, the inserts were washed with phenol red-free DMEM (Thermo Fisher Scientific) and placed in 24-well tissue culture plates containing 400μL of phenol red-free DMEM/10% FBS in each well. Dextran-FITC (125 μg/mL, 70kDa, Sigma) (200μL) was added to the top of the insert, and after 5 min at 37°C, media was collected from the lower chamber as a measure of permeability. Fluorescence was quantified with a fluorescence-detecting plate reader (excitation λ 488 nm; emission λ 510 nm).

### Flow cytometry

After treatment with sTREM2, astrocytes were detached using a Trypsin/EDTA mix. The cells were then washed, resuspended in PBS with 2% BSA and centrifuged twice at 1800 rpm for 5 min at 4°C. Cells were resuspended in PBS and incubated for 15 min with 4nM DAPI to label dead cells. The FIX/PERM Kit was used for intracellular staining with antibodies to GFAP (Cell Signaling #3655S), p65 p-NFKB at Ser536 (Cell Signaling #5733). Labeled cells were fixed with 2% paraformaldehyde in PBS and analyzed using a flow cytometer NovoCyte Quanteon Flow Cytometer Systems 4 Lasers).

### Immunofluorescence

Primary human astrocytes were seeded in 24-well plates onto glass coverslips (Poly-D-Lysine Cellware 12 mm round coverslips #354086, Corning), at 20,000 cells/well and grown for 24 hrs. After treatment, cells were fixed in 4% paraformaldehyde for 20 min and permeabilized in 0.01% Triton x-100 for 5 min. Coverslips were blocked for 1 hour in block solution (3% essentially immunoglobulin-free BSA (Sigma) in PBS) for 30 min. They were then incubated overnight at 4°C with anti-GFAP antibody (#3670s, Cell signaling technology) at 1:800 in block solution. After washing with PBS, the coverslips were incubated with secondary antibody Alexa Fluor 594 goat anti mouse antibody (#A11005, Invitrogen) diluted 1:1000 and also labeled with phalloidin Alexa Fluor 488 (#A12379Invitrogen) diluted 1:400. Coverslips were mounted in Prolong Diamond Antifade Mountant with DAPI (#P36962, Invitrogen) and dried at room temperature for 24 h. Cells were visualized by the Correlative Light and Electron Microscopy *Zeiss Axio Observer* by an observer blinded to the treatment condition. Fifty to 70 cells per condition were analyzed in ImageJ for the fluorescent intensity of GFAP (Red) per cell. Median fluorescence per treatment was averaged for each experiment, converted to a fold change of GFAP intensity relative to the untreated control set to 1.0, and tested for statistical significance.

### Immunofluorescence of BBB membranes.

BBB membranes were analyzed by immunostaining for Zona occludens-1 (ZO-1). Inserts were fixed with in 4% paraformaldehyde for 40 min and permeabilized in 0.01% Triton x-100 for 15 min. The inserts were blocked for 1 hour in 3% BSA/PBS. Inserts were incubated overnight at 4°C in primary anti- ZO-1 antibody (ZO-1 (D6L1E) Rabbit Monoclonal Antibody #13663, cell signaling) diluted 1:400 or Isotype control antibody (Rabbit IgG, #3900S, cell signaling). After three washes with 1% BSA/PBS, inserts were incubated with Alexa Fluor^™^ 488 goat anti-rabbit IgG secondary antibody (Life Technologies, A11008) diluted 1:2000 in 3% BSA/PBS for 1h. Actin filaments were stained simultaneously with Alexa Fluor^™^ 647 phalloidin (Invitrogen, A22287) diluted 1:400 in 3% BSA/PBS for 20 min at room temperature. After watching with 1% BSA in PBS the membrane of the inserts was carefully cut out and mounted in Prolong Diamond Antifade Mountant with DAPI (Invitrogen) and dried overnight. The preparation was observed on a confocal microscope (Bio-Rad Radiance 2000 laser scanning confocal microscope) and photographed. The fluorescence is observed using an inverted confocal microscope equipped (Nikon, Melville, NY,USA).

### Multiplex Assay

Cytokines and MMP were measured in supernatants of treated astrocytes using single analyte ELISAs and multiplex bead-based assays. For the metalloproteinases ProcartaPlex^™^ Human MMP Panel 2, 3 plex (Thermofisher # EPX03A-10829-901) and MMP-2 Human ProcartaPlex^™^ Simplex Kit (Thermofisher # EPX01B-12132-901) were used. Assays were performed according to manufacturer’s recommended protocols. Data was collected on a Bio-Plex 200 system (Bio-Rad) and analyzed using Bio-Plex manager software. Samples were assayed in duplicate. Assay ranges were as specified by the manufacturers. For cytokines, the Human Inflammation Panel 1 (LEGENDplex^™^ Cat. No. 740808 Human Inflammation Panel 1) was used. Data was acquired with the NovoCyte Quanteon Flow Cytometer Systems 4 Lasers, and was analyzed with online software provided by Biolegend (biolegend.com/en-us/legendplex).

### Statistics

Values were normalized to the control (vehicle) within each experiment and analyzed using t-tests against a theoretical mean of 1. A p < 0.05 is significant. Analyses were performed using GraphPad Prism.

## Results

### sTREM2 promotes a reactive state in astrocytes

**Figure 1:**
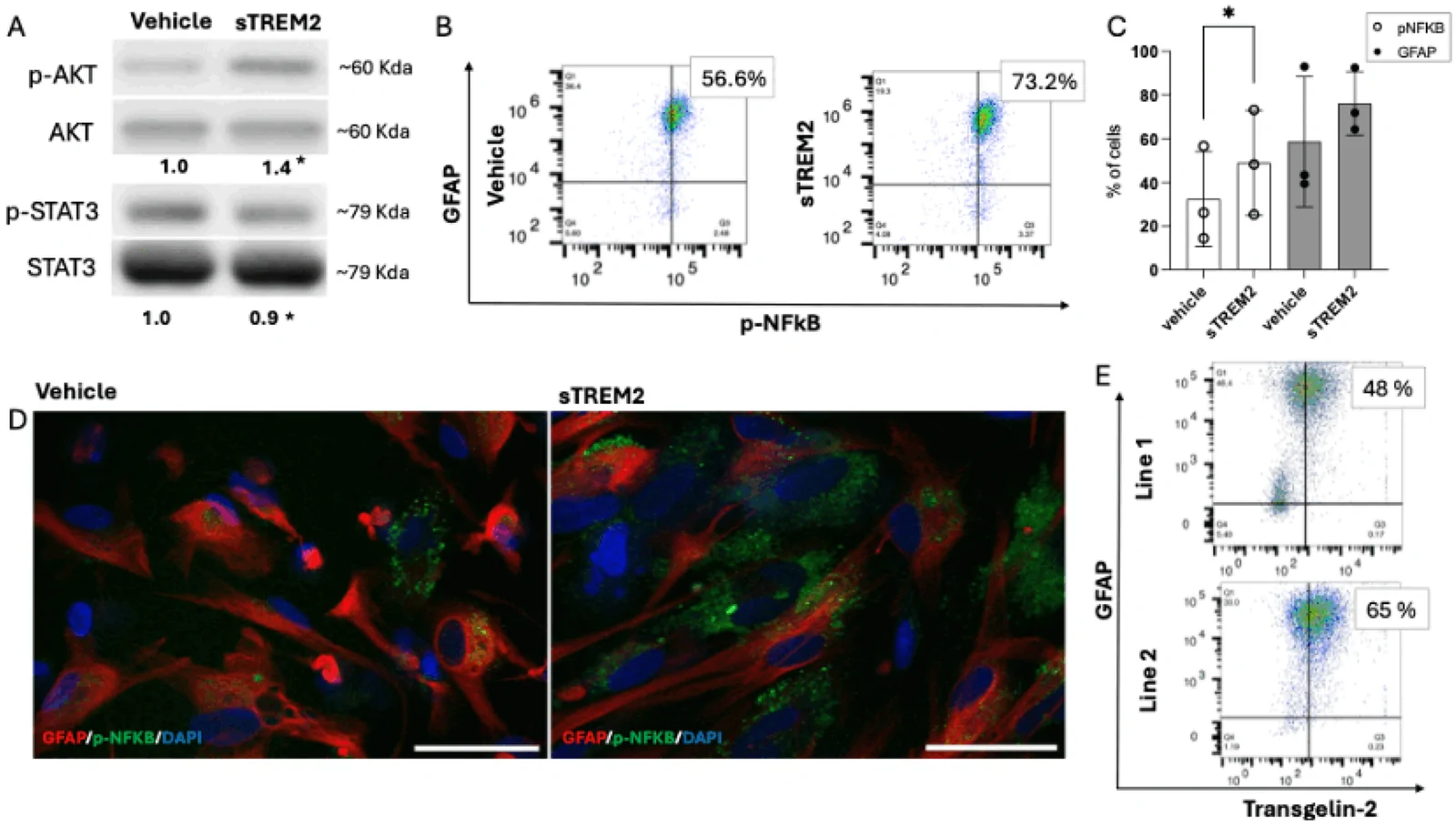
Pathways associated with astrocyte reactivity in response to sTREM2. (A) Levels of AKT (p-AKT ser473) (n = 4) and phosphorylated STAT3 (p-STAT3) (n = 5) in astrocytes after 6 h incubation with 100 nM sTREM2. (B) Flow cytometry dot plot of astrocytes treated with 100 nM sTREM2 for 6 h showing GFAP and p-NFkB levels (n=3). (C) Flow cytometry quantification of astrocytes treated with 100 nM sTREM2 for 6 h showing GFAP and p-NFkB levels (D) Confocal image showing p-NFkB (green), GFAP (red), and nuclei (blue) in astrocytes treated with 100 nM sTREM2 (n = 2). Scale bar for all images: 50 μm. (E) Flow cytometry dot plot of two different astrocyte lineages showing GFAP and Transgelin-2 levels (n=2). Data are presented as mean ± SEM. *p ≤ 0.05.

Under inflammatory conditions, astrocytes shift from their classical phenotype to an inflammatory phenotype and are referred to as reactive astrocytes (RAs)^34^. This state is characterized by expression of inflammatory molecules that sustain neuroinflammation within the CNS^35^. The production and release of these molecules are regulated by signaling pathways including STAT3^36^, PI3K/AKT^37,38^, and NF-κB^39–41^. To characterize the role of sTREM2 in astrocyte reactivity, we examined the activation of these key signaling pathways. Astrocytes were treated with 100nM sTREM2 for 6 h. To define the temporal dynamics of sTREM2 signaling, we performed a kinetic analysis across multiple time points. Full uncropped Western blot images demonstrating maximal AKT phosphorylation following 6 h of sTREM2 treatment are provided in Figure S1. There was an ~ 44% increase in p-AKT levels compared with vehicle-treated controls (Fig. 1A). Quantification of the data presented in Figure S2. In contrast, STAT3 phosphorylation was slightly reduced relative to controls (Fig. 1A). Full uncropped Western blot images of STAT3 phosphorylation provided in Figure S3.

We next analyzed the expression of NFkB and GFAP by flow cytometry. Treatment with sTREM2 elevated p-NFkB compared to control (Fig. 1B and C), comparable to the levels inducted by TNF-a (Fig.S4). There were no clear differences in the percentage of GFAP-positive cells but microscopic analyses showed an increase in GFAP in cells incubated with sTREM2. The gating strategy for flow cytometry is detailed in (Fig. S4). However, GFAP expression detected by microscopy showed significant differences under the same conditions, supporting that sTREM2 increases GFAP expression in astrocytes (Fig. S5). Additionally, there was increased p-NFkB in astrocytes treated with sTREM, shown by microscopy, consistent with the elevated levels detected by flow cytometry (Fig. 1D). These findings suggest that sTREM2 promotes astrocyte reactivity through activation of the PI3K/AKT and NFkB pathways.

We then examined potential receptors mediating sTREM2 signaling in astrocytes. While no sTREM2 receptor has been described in astrocytes, we assessed the expression of a recently identified neuronal sTREM2 receptor, Transgelin-2 (TAGLN2)^13^. Surface expression of this receptor was detected by flow cytometry in two independent primary astrocyte (Line 1 and Line 2), supporting its potential involvement in astrocytic sTREM2 responses (Fig. 1E). The gating strategy is detailed in (Fig. S6).

### sTREM2 increases the permeability of the BBB

**Figure 2:**
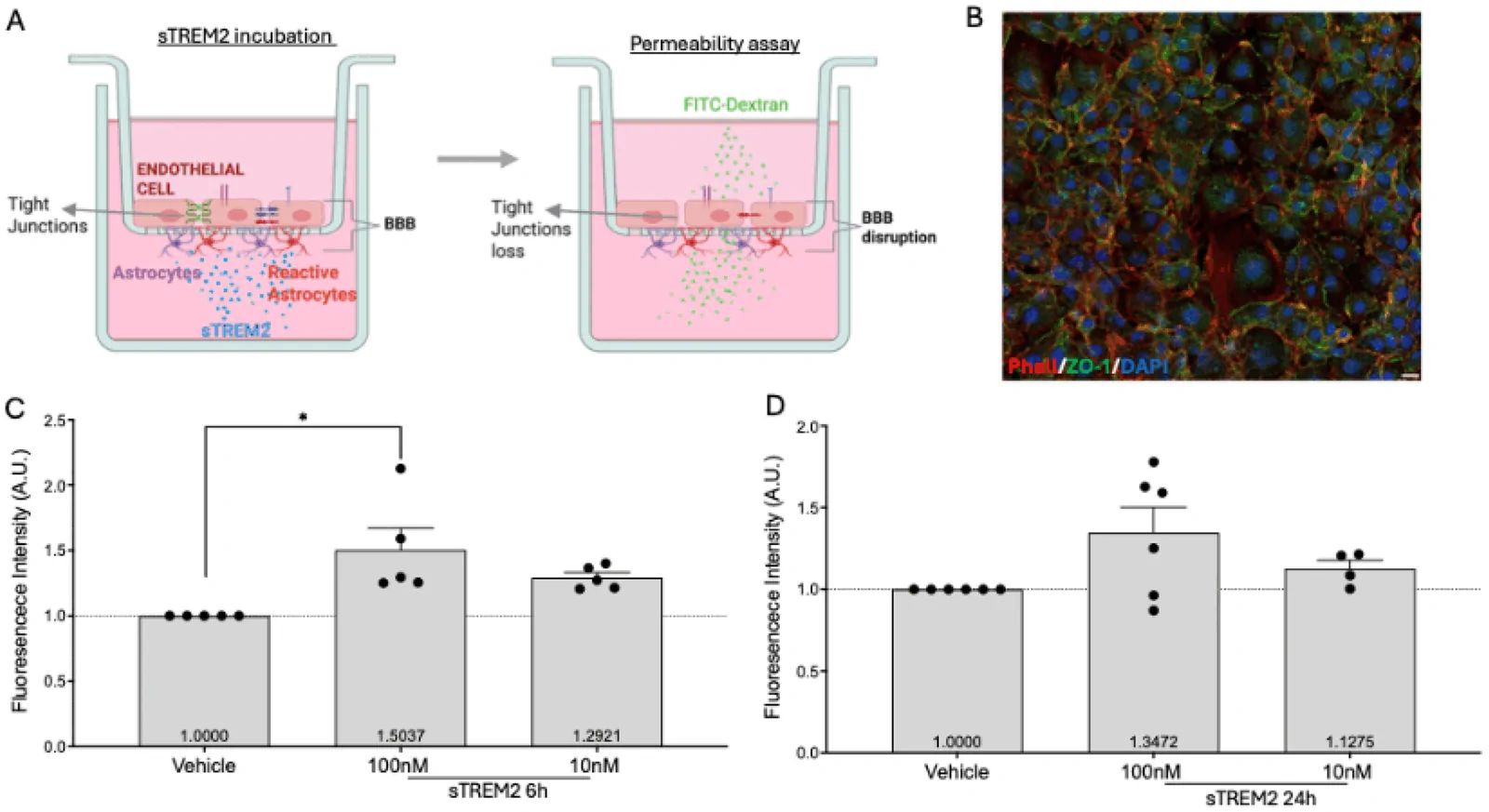
Permeability assay of an *in vitro* human BBB model in presence of sTREM2. (A) Schematic representation of the human BBB model, illustrating its components and the permeability assay. The left panel shows a monolayer of BMVEC in the upper compartment with astrocytes growing on the opposite side of the semipermeable membrane. These astrocytes extend foot processes through the pores of the membrane contacting the endothelium^32^. During sTREM2 incubation, astrocytes become reactive. The right panel shows the permeability assay. After sTREM2 treatment, the medium was replaced, and FITC–dextran added to the upper compartment. Increased fluorescence in the lower compartment indicates BBB permeability, likely due to loss of tight junctions mediated by RAs. (B) Representative microscopy image of the endothelial cell monolayer in the upper compartment stained with phalloidin (red), ZO-1 (green), and DAPI (blue). Scale bar: 20 μm. (C, D) Quantification of fluorescence intensity detected in the lower chamber following BBB incubation with 100 nM or 10 nM sTREM2 for 6 h (C) or 24 h (D). Data are presented as mean ± SEM. *p ≤ 0.05.

Increased BBB permeability has been reported in several neurological diseases^42^, and astrocytes play a central role in maintaining BBB integrity and function^43^. To determine whether sTREM2 affected BBB permeability through astrocyte reactivity, we used a human in vitro co-culture BBB model, which has been extensively used to study mechanisms of PBMC transmigration^16–24^. This model consists of primary BMVEC and primary human cortical astrocytes cultured on opposite sides of a semipermeable transwell membrane (Fig. 2A). Soluble TREM2 (100 or 10 nM) was added to the astrocytic compartment (bottom, CNS side) for 6 h or 24 h. Following treatment, BBB permeability was assessed using a dextran-FITC assay (Fig. 2A). An image of the BBB BMVEC shows a compact monolayer with tight junctions, with ZO-1 immunostaining outlining intercellular borders of the cell (Fig. 2B).

The permeability assay showed a significant increase in permeability in sTREM2 treated BBB cultures at 6 h with 100 nM, but not with 10 nM (Fig. 2C). A similar trend was found at 24 h, where 100 nM sTREM2 increased permeability; however, at this time the differences are not statistical significance compared with untreated controls (Fig. 2D). These findings suggest that sTREM2 promotes BBB permeability, likely through astrocyte reactivity. To address this further, we examined the mechanisms by which sTREM2 mediated permeability.

### sTREM2 disrupts the tight junctions of endothelial cells of the BBB

BBB permeability is a dynamic process that regulates the entry and exit of molecules from the CNS^44^. BMVEC are central to this regulation, as they control barrier permeability, in part through the expression of TJ proteins^45^. Previous studies showed that astrocytes can modulate BBB permeability by regulating endothelial TJ^43,46,47^. When astrocytes adopt a reactive phenotype, they can disrupt this balance, leading to the loss of endothelial TJs and impaired barrier integrity^43,46,47^.

**Figure 3:**
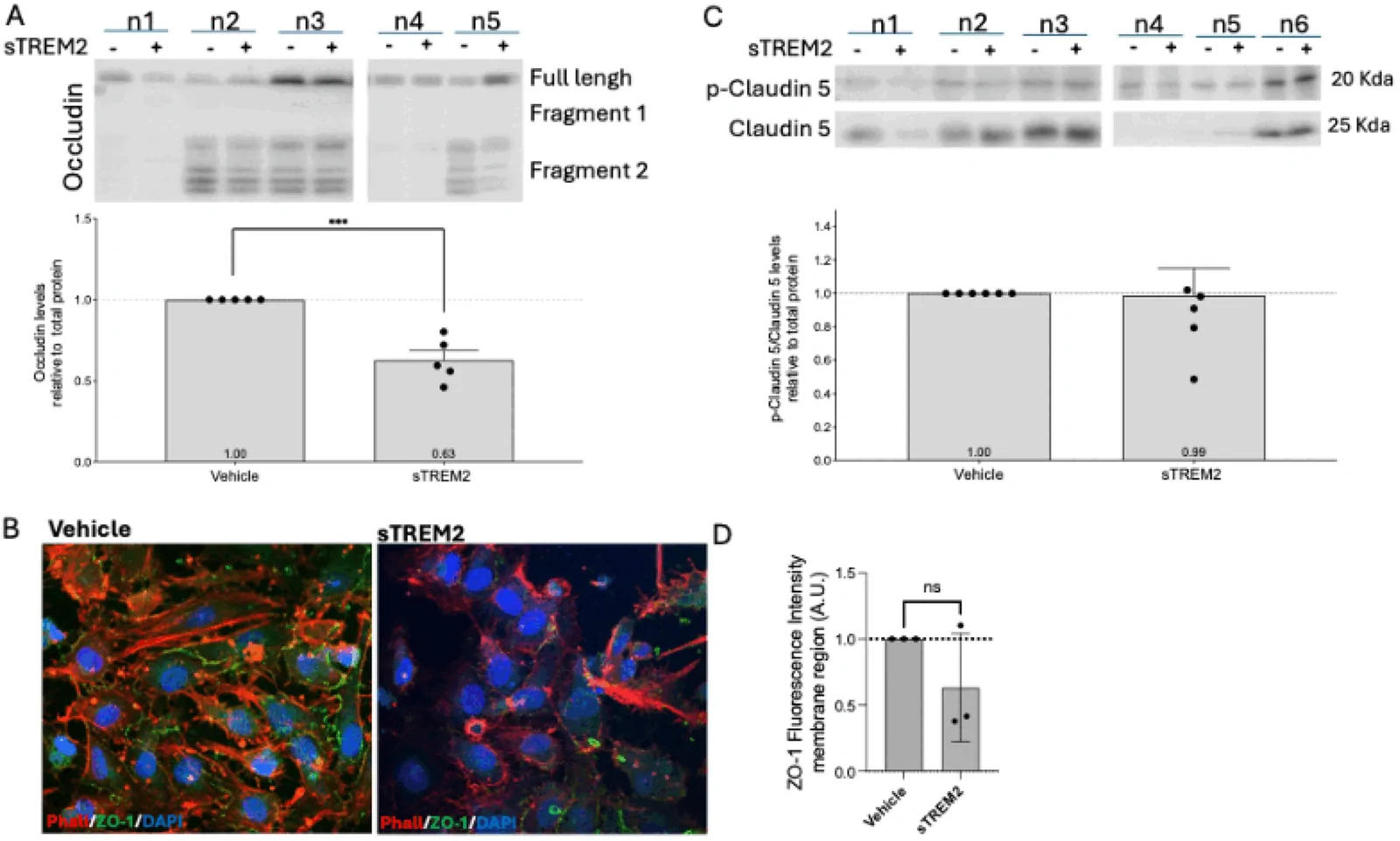
Tight junction expression and distribution changes in response to sTREM2. (A) Analysis of occludin in astrocytes (n = 5) treated with 100 nM sTREM2. The upper panel shows full-length occludin (~65–67 kDa) and its fragments 1 (~50–55 kDakDa) and 2 (~37–42 kDa). The accompanying graph quantifies occludin expression relative to total protein and normalized to vehicle control. (B) Analysis of phosphorylated claudin-5 and total claudin-5 expression in astrocytes (n = 5) treated with 100 nM sTREM2. The graph shows the quantified p–claudin-5/total claudin-5 ratio relative to total protein and normalized to vehicle. (C) Representative image of BBB BMVECtreated with 100 nM sTREM2 or vehicle for 6 h in the bottom chamber, stained with phalloidin (red), ZO-1 (green), and DAPI (blue) (n = 3). (D) Analysis of ZO-1 fluorescence intensity at the cell-cell contact area (normalized per cell) in astrocytes treated with 100 nM sTREM2 or vehicle for 6h (n = 3). ***p ≤ 0.001. Scale bar for all images: 50 μm.

To examine how sTREM2 affects BBB permeability, we analyzed TJ proteins in the endothelial monolayer. After incubating the BBB with sTREM2 applied only to the astrocytic compartment, BMVEC were collected from the opposite side of the insert by scraping. Proteins isolated from BMVEC collected from BBB model were analyzed by Western blotting, demonstrating a significant reduction in Occludin following sTREM2 treatment (Fig. 3A). Full uncropped Western blot images for Occludin and total protein loading control are provided are shown in (see Fig. S7).

(A) Analysis of occludin in astrocytes (n = 5) treated with 100 nM sTREM2. The upper panel shows full-length occludin (~ 65–67 kDa) and its fragments 1 (~ 50–55 kDakDa) and 2 (~ 37–42 kDa). The accompanying graph quantifies occludin expression relative to total protein and normalized to vehicle control. (B) Analysis of phosphorylated claudin-5 and total claudin-5 expression in astrocytes (n = 5) treated with 100 nM sTREM2. The graph shows the quantified p–claudin-5/total claudin-5 ratio relative to total protein and normalized to vehicle. (C) Representative image of BBB BMVEC treated with 100 nM sTREM2 or vehicle for 6 h in the bottom chamber, stained with phalloidin (red), ZO-1 (green), and DAPI (blue) (n = 3). (D) Analysis of ZO-1 fluorescence intensity at the cell-cell contact area (normalized per cell) in astrocytes treated with 100 nM sTREM2 or vehicle for 6h (n = 3). ***p ≤ 0.001. Scale bar for all images: 50 μm.

The analysis and quantification detected fragments derived from the protein, ranging from the full-length form (63 kDa) to fragment 2 (31 kDa) described previusly^48^. In contrast, Claudin-5, a TJ protein that requires phosphorylation for membrane function, showed no change in the p-Claudin-5/Claudin-5 ratio (Fig. 3B). Full uncropped Western blot images for p–claudin-5/total claudin-5 are provided are shown in (Fig. S8). Immunofluorescence analysis of ZO-1 demonstrated a redistribution pattern, with reduced localization at cell–cell junctions in the presence of sTREM2 (Fig. 3C). However, quantification of ZO-1 expression at the junctional area did not reach statistical significance, presumably due to high variability (Fig. 3D).

Together, these findings indicate that sTREM2 disrupts endothelial TJ expression and distribution, which may contribute to the increased BBB permeability found with sTREM2 treatment.

### sTREM2 induces astrocyte secretion of MMP2

**Figure 4:**
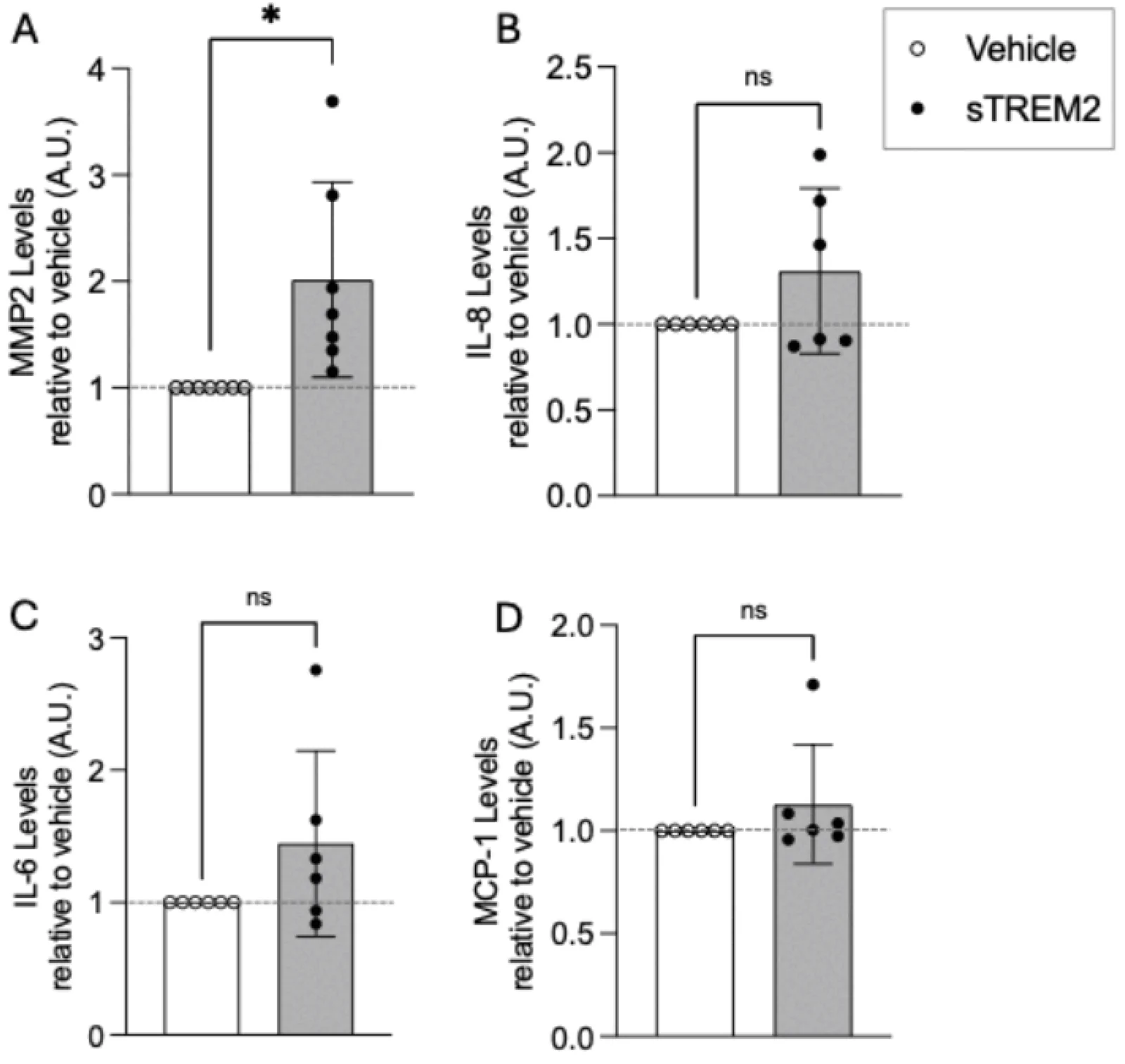
Analysis of inflammatory mediators detected in supernatants from primary astrocytes treated with sTREM2 or vehicle conditions for 24 h. including (A) MMP2 (n=7) *p ≤ 0.05. , (B) IL-8 (n=7) p = 0.14, (C) IL-6 (n=7) p = 0.15 and (D) MCP-1(n=7) p = 0.3.

Astrocyte derived soluble factors, including cytokines, have been implicated in BBB disruption in multiple pathological contexts^43,49^. MMPs contributes to Occludin degradation, leading to BBB dysfunction and neurological damage^50^. To examine whether sTREM2 causes MMP release by astrocytes, we analyzed the supernatant of sTREM2-treated astrocytes treated for 24 hours with vehicle or sTREM2 using multiplex ELISA for MMP-3, MMP-9 and MMP2. Our analysis showed that MMP-2 was significantly increased in the sTREM2 condition (Fig. 4A). MMP-3 and MMP-9 were undetectable in all conditions (Below Lower Limit of Quantification), see Fig. S9. These results identify MMP-2 as a potential astrocyte-derived mediator contributing to the increased BBB permeability among the MMPs analyzed. Additionally, to identify cytokines candidate mediators involved in this process, we performed a commercial multiplex cytokine analysis including inflammatory factors, such as TNF-a, IL-1b, IL-18, IL-33, IL-8, IL-12pa70, and MCP-1. These have been found to be associated with BBB disruption in various studies^51–58^.

Only IL-8, IL-6 and MCP-1 were detected at levels above Lower Limit of Quantification. (see Table S1). Although not reaching statistical significance, IL-8 (Fig. 4B), IL-6 (Fig. 4C) and MCP-1(Fig. 4D) showed consistent upward trends following sTREM2 exposure.

These findings suggest that sTREM2 may promote the release of molecules and cytokines involved in BBB disruption, with MMP2 potentially serving as a key mediator of this process.

## Discussion

TREM2 has been extensively examined in immunology and neuroscience studies. However, the effects of the released soluble form, sTREM2, are less well characterized. sTREM2 has been implicated as a promoter of inflammation. However, in other studies it reduces inflammation, suggesting a dual role rather than a uniformly beneficial or detrimental effect^59,60^. In this work, we examined the impact of sTREM2 on BBB permeability by analyzing its effects on astrocytes, a major component of the barrier.

Currently, there are no published studies addressing the impact of sTREM2 on astrocytes. To our knowledge, the results in this study show, for the first time, molecular pathways associated with human astrocytes in response to sTREM2 treatment and their impact in BBB integrity.

The three key molecules most associated with astrocytic reactivity are p-NFkB^39,41,61^, p-AKT^38,62^, and STAT3^46,62,63^.Thus, we analyzed the activation status of these pathways and found that sTREM2 primarily induces activation of p-AKT and p–NF-κB. This correlates with increased expression of GFAP, a hallmark of RAs, often correlated with changes in sTREM2 levels^64,65^, supporting that sTREM2 promotes astrocyte reactivity through these signaling pathways. The increased phosphorylation of NF-κB found after sTREM2 treatment was consistent with the enhanced astrocyte reactivity detected in our study, supporting activation of aninflammatory phenotype. Although STAT3 signaling is traditionally associated with reactive astrogliosis, evidence suggests that its activation may also decrease in certain inflammatory contexts ^66–68^. Alternatively, while sustained NF-κB signaling may contribute to maintaining the inflammatory phenotype, the reduction in p-STAT3 may reflect a compensatory response following an initial activation phase during early astrocyte activation. Further studies examining STAT3 activation dynamics and nuclear localization at earlier time points will be required to clarify the role of STAT3 in sTREM2-induced astrocyte reactivity.

The impact of sTREM2 also showed a significant decrease in Occludin protein levels in BMVEC forming the BBB, suggesting that astrocyte reactivation induced by sTREM2 may underlie this reduction. Occludin has been associated with disruption in of BBB integrity and neurological function after ischemic stroke in mice^69^. Blood levels of soluble Occludin l have been proposed as a potential biomarker for early BBB damage following ischemic stroke^70^. Aditionally, has been implicated in facilitating pathogen entry into the CNS^71,72^ and has been proposed as a potential therapeutic target to limit viral invasion of CNS^73^. We found a significant increase in MMP2 after sTREM2 treatment, consistent with previous studies that demonstrated MMP2 increases BBB permeability in in vitro models^74^. This is also consistent with previous reports showing that MMP2 mediates Occludin degradation and BBB damage during the early stages of ischemic stroke ^75^. Thus, our suggest a key role of MMP2 as a potential responsible of the BBB disruption produced by sTREM2 through occluding degradation of the BBB. Although IL-18, IL-6 and MCP-1 were the only cytokines consistently detectable amongst the selected analytes in the supernatant, we did not observe statistically significant increases following sTREM2 treatment. However, both cytokines showed a consistent upward trend, suggesting a potential involvement in BBB dysfunction. With respect to the other tight junction proteins analyzed, although ZO-1 changes were not statistically significant, two of the three BBB preparations showed marked alterations of ZO-1 following sTREM2 exposure, suggesting a potential effect on tight junction integrity that should be further investigated.

A limitation of this study is that other MMPs and cytokines measured, although not detected or detected at very low levels by our assay, could still be of significance in BBB disruption. Given the proximity of astrocytes to BMVEC, only small amounts of these molecules, presumably undetectable by ELISA, may be sufficient to affect TJ integrity. Thus, their contribution cannot be excluded.

We also demonstrated TAGLN2 expression in astrocytes. Given its recent identification as a sTREM2 receptor in neurons, this raises the possibility that TAGLN2 may participate in sTREM2 signaling in astrocytes^13^. However, most publications describe TAGLN2 as a cytosolic actin-binding protein that lacks a signal peptide or transmembrane domain and is mainly localized to membrane associated cytoskeletal structures rather than the extracellular plasma membrane^76^. Therefore, detection of TAGLN2 in non permeabilized cells, including by flow cytometry, is consistent with a membrane proximal localization, but does not necessarily indicate that TAGLN2 functions as a transmembrane receptor with an extracellular domain. Additional studies are needed to confirm whether this protein functions as such a receptor in astrocytes BBB permeability.

## Conclusion

In this study, we characterized the impact of sTREM2 on the integrity of the BBB using a human in vitro model of primary human BMVEC and primary human astrocytes. We showed that sTREM2 promoted an inflammatory state characterized by the activation of pathways consistent with reactivity in astrocytes. We detected a decrease in the TJ protein Occludin that may contribute to loss of permeability and correlates with elevated MMP2 release from astrocytes, suggesting a potential mechanism of sTREM2-mediated BBB disruption. Our findings support a role for sTREM2-mediated astrocyte reactivity in BBB dysfunction and highlight potential therapeutic targets for neurocognitive disorders associated with BBB disruption.

## Supplementary Material

This is a list of supplementary files associated with this preprint. Click to download.

- SupplementaryFile.pdf

## Data Availability

The datasets generated and/or analyzed during the current study are available from the corresponding author on reasonable request.

## Abbreviations

sTREM2: Soluble TREM2
BBB: Blood Brain Barrier
CNS: Central Nervous System
RA: Reactive astrocyte
EC: Endothelial Cells
BMVECs: Brain Microvascular Endothelial Cells
MMP: Matrix metalloproteinases
NFkB: Nuclear Factor kappa-light-chain-enhancer of activated B cells
p-NFkB: Phospo-Nuclear Factor kappa-light-chain-enhancer of activated
STAT3: Signal Transducer and Activator of Transcription 3
pSTAT3: Phospo- Signal Transducer and Activator of Transcription 3
AKT: Ak third kinase
pAKT: Phospho-Ak third kinase
ZO-1: Zonula occludens-1
TJ: Tigh Junction
TAGLN2: Transgelin-2

## References

[1] Ulland TK, Colonna M. TREM2 — a key player in microglial biology and Alzheimer disease. Nature Reviews Neurology. 2018;14(11):667–675. 30266932 10.1038/s41582-018-0072-1

[2] Pocock J, Vasilopoulou F, Svensson E, Cosker K. Microglia and TREM2. Neuropharmacology. 2024;257:110020. 38821351 10.1016/j.neuropharm.2024.110020

[3] Li Y, Xu H, Wang H, Yang K, Luan J, Wang S. TREM2: Potential therapeutic targeting of microglia for Alzheimer’s disease. Biomedicine & Pharmacotherapy. 2023;165:115218. 37517293 10.1016/j.biopha.2023.115218

[4] Deczkowska A, Weiner A, Amit I. The Physiology, Pathology, and Potential Therapeutic Applications of the TREM2 Signaling Pathway. Cell. 2020;181(6):1207–1217. 32531244 10.1016/j.cell.2020.05.003

[5] Lopez-Ortiz AO, Doceti M, Thomas J, Transcriptional Regulation of Microglial Metabolic and Activation States by P2RY12. Glia. 2025;73(12):2464–2482. 40814757 10.1002/glia.70078 PMC12541897

[6] Brown GC, St George-Hyslop P. Does Soluble TREM2 Protect Against Alzheimer’s Disease? Front Aging Neurosci. 2021;13:834697. 35153729 10.3389/fnagi.2021.834697 PMC8831327

[7] Suárez-Calvet M, Morenas-Rodríguez E, Kleinberger G, Early increase of CSF sTREM2 in Alzheimer’s disease is associated with tau related-neurodegeneration but not with amyloid-β pathology. Molecular Neurodegeneration. 2019;14(1):1. 30630532 10.1186/s13024-018-0301-5 PMC6327425

[8] Suárez-Calvet M, Kleinberger G, Araque Caballero M, sTREM2 cerebrospinal fluid levels are a potential biomarker for microglia activity in early-stage Alzheimer’s disease and associate with neuronal injury markers. EMBO Mol Med. 2016;8(5):466–476. 26941262 10.15252/emmm.201506123 PMC5120370

[9] Bekris LM, Khrestian M, Dyne E, Soluble TREM2 and biomarkers of central and peripheral inflammation in neurodegenerative disease. J Neuroimmunol. 2018;319:19–27. 29685286 10.1016/j.jneuroim.2018.03.003 PMC6036902

[10] Wang Y, Ye M, Ji Q, Liu Q, Xu X, Zhan Y. Association between Cerebrospinal Fluid sTREM2 Levels and Depression: The Alzheimer’s Disease Neuroimaging Initiative Study. J Prev Alzheimers Dis. 2024;11(4):1087–1092. 39044521 10.14283/jpad.2024.70 PMC12275841

[11] Zhong L, Xu Y, Zhuo R, Soluble TREM2 ameliorates pathological phenotypes by modulating microglial functions in an Alzheimer’s disease model. Nature Communications. 2019;10(1):1365. 10.1038/s41467-019-09118-9 PMC6433910 30911003

[12] Franzmeier N, Suárez-Calvet M, Frontzkowski L, Higher CSF sTREM2 attenuates ApoE4-related risk for cognitive decline and neurodegeneration. Molecular Neurodegeneration. 2020;15(1):57. 33032659 10.1186/s13024-020-00407-2 PMC7545547

[13] Zhang X, Tang L, Yang J, Soluble TREM2 ameliorates tau phosphorylation and cognitive deficits through activating transgelin-2 in Alzheimer’s disease. Nature Communications. 2023;14(1):6670. 10.1038/s41467-023-42505-x PMC10590452 37865646

[14] Winfree RL, Dumitrescu L, Blennow K, Biological correlates of elevated soluble TREM2 in cerebrospinal fluid. Neurobiol Aging. 2022;118:88–98. 35908327 10.1016/j.neurobiolaging.2022.06.013 PMC9707345

[15] Li R-Y, Qin Q, Yang H-C, TREM2 in the pathogenesis of AD: a lipid metabolism regulator and potential metabolic therapeutic target. Molecular Neurodegeneration. 2022;17(1):40. 35658903 10.1186/s13024-022-00542-y PMC9166437

[16] Williams DW, Calderon TM, Lopez L, Mechanisms of HIV entry into the CNS: increased sensitivity of HIV infected CD14 + CD16+ monocytes to CCL2 and key roles of CCR2, JAM-A, and ALCAM in diapedesis. PLoS One. 2013;8(7):e69270. 23922698 10.1371/journal.pone.0069270 PMC3724935

[17] Veenstra M, Williams DW, Calderon TM, Anastos K, Morgello S, Berman JW. Frontline Science: CXCR7 mediates CD14(+)CD16(+) monocyte transmigration across the blood brain barrier: a potential therapeutic target for NeuroAIDS. J Leukoc Biol. 2017;102(5):1173–1185. 28754798 10.1189/jlb.3HI0517-167R PMC5636044

[18] Buckner CM, Calderon TM, Willams DW, Belbin TJ, Berman JW. Characterization of monocyte maturation/differentiation that facilitates their transmigration across the blood-brain barrier and infection by HIV: implications for NeuroAIDS. Cell Immunol. 2011;267(2):109–123. 21292246 10.1016/j.cellimm.2010.12.004 PMC4335637

[19] Williams DW, Eugenin EA, Calderon TM, Berman JW. Monocyte maturation, HIV susceptibility, and transmigration across the blood brain barrier are critical in HIV neuropathogenesis. J Leukoc Biol. 2012;91(3):401–415. 22227964 10.1189/jlb.0811394 PMC3289493

[20] Veenstra M, León-Rivera R, Li M, Gama L, Clements Janice E, Berman Joan W. Mechanisms of CNS Viral Seeding by HIV+ CD14 + CD16+ Monocytes: Establishment and Reseeding of Viral Reservoirs Contributing to HIV-Associated Neurocognitive Disorders. mBio. 2017;8(5):10.1128/mbio.01280-01217. PMC5654927 29066542

[21] Eugenin EA, Osiecki K, Lopez L, Goldstein H, Calderon TM, Berman JW. CCL2/Monocyte Chemoattractant Protein-1 Mediates Enhanced Transmigration of Human Immunodeficiency Virus (HIV)-Infected Leukocytes across the Blood–Brain Barrier: A Potential Mechanism of HIV–CNS Invasion and NeuroAIDS. The Journal of Neuroscience. 2006;26(4):1098–1106. 16436595 10.1523/JNEUROSCI.3863-05.2006 PMC6674577

[22] León-Rivera R, Veenstra M, Donoso M, Central Nervous System (CNS) Viral Seeding by Mature Monocytes and Potential Therapies To Reduce CNS Viral Reservoirs in the cART Era. mBio. 2021;12(2). 10.1128/mBio.03633-20 PMC8092320 33727362

[23] Veksler V, Leon-Rivera R, Fleysher L, CD14 + CD16+ monocyte transmigration across the bloodbrain barrier is associated with HIV-NCI despite viral suppression. JCI Insight. 2024;9(17). 10.1172/jci.insight.179855 PMC11385088 39253970

[24] Ruiz VY, Calderon TM, Leon-Rivera R, Chilunda V, Zhang J, Berman JW. Single-cell analysis of CD14(+)CD16(+) monocytes identifies a subpopulation with an enhanced migratory and inflammatory phenotype. Front Immunol. 2025;16:1475480. 40051633 10.3389/fimmu.2025.1475480 PMC11883828

[25] Eugenin EA, D’Aversa TG, Lopez L, Calderon TM, Berman JW. MCP-1 (CCL2) protects human neurons and astrocytes from NMDA or HIV-tat-induced apoptosis. J Neurochem. 2003;85(5):1299–1311. 12753088 10.1046/j.1471-4159.2003.01775.x

[26] Hurwitz AA, Lyman WD, Guida MP, Calderon TM, Berman JW. Tumor necrosis factor alpha induces adhesion molecule expression on human fetal astrocytes. J Exp Med. 1992;176(6):1631–1636. 1281214 10.1084/jem.176.6.1631 PMC2119454

[27] Weiss JM, Downie SA, Lyman WD, Berman JW. Astrocyte-derived monocyte-chemoattractant protein-1 directs the transmigration of leukocytes across a model of the human blood-brain barrier. J Immunol. 1998;161(12):6896–6903. 9862722

[28] Weiss JM, Berman JW. Astrocyte expression of monocyte chemoattractant protein-1 is differentially regulated by transforming growth factor beta. J Neuroimmunol. 1998;91(1–2):190–197. 9846835 10.1016/s0165-5728(98)00183-0

[29] Hurwitz AA, Berman JW, Rashbaum WK, Lyman WD. Human fetal astrocytes induce the expression of blood-brain barrier specific proteins by autologous endothelial cells. Brain Res. 1993;625(2):238–243. 7903899 10.1016/0006-8993(93)91064-y

[30] Lee SC, Liu W, Dickson DW, Brosnan CF, Berman JW. Cytokine production by human fetal microglia and astrocytes. Differential induction by lipopolysaccharide and IL-1 beta. J Immunol. 1993;150(7):2659–2667. 8454848

[31] Cheney L, McDermott G, Guzik H, Berman JW. Antiretroviral Drugs Impact Autophagy Differently in Primary Human Astrocytes. Cells. 2025;14(23). 10.3390/cells14231904 PMC12691388 41369393

[32] Eugenin EA, Clements JE, Zink MC, Berman JW. Human immunodeficiency virus infection of human astrocytes disrupts blood-brain barrier integrity by a gap junction-dependent mechanism. J Neurosci. 2011;31(26):9456–9465. 21715610 10.1523/JNEUROSCI.1460-11.2011 PMC3132881

[33] Wu MC, Hsu JL, Lai TW. Evans blue dye as an indicator of albumin permeability across a brain endothelial cell monolayer in vitro. Neuroreport. 2021;32(11):957–964. 34227616 10.1097/WNR.0000000000001690

[34] Patani R, Hardingham GE, Liddelow SA. Functional roles of reactive astrocytes in neuroinflammation and neurodegeneration. Nature Reviews Neurology. 2023;19(7):395–409. 37308616 10.1038/s41582-023-00822-1

[35] Zhao Y, Huang Y, Cao Y, Yang J. Astrocyte-Mediated Neuroinflammation in Neurological Conditions. Biomolecules. 2024;14(10). 10.3390/biom14101204 PMC11505625 39456137

[36] Cao Y, Lin X, Gao D, Yang J, Miao H, Li T. Inhibition of STAT3 phosphorylation attenuates perioperative neurocognitive disorders in mice with D-galactose-induced aging by regulating pro-inflammatory reactive astrocytes. Int Immunopharmacol. 2025;148:114095. 39827670 10.1016/j.intimp.2025.114095

[37] Cheng YY, Ding YX, Bian GL, Reactive Astrocytes Display Pro-inflammatory Adaptability with Modulation of Notch-PI3K-AKT Signaling Pathway Under Inflammatory Stimulation. Neuroscience. 2020;440:130–145. 32450294 10.1016/j.neuroscience.2020.05.023

[38] Pérez-Núñez R, González MF, Avalos AM, Leyton L. Impacts of PI3K/protein kinase B pathway activation in reactive astrocytes: from detrimental effects to protective functions. Neural Regen Res. 2025;20(4):1031–1041. 38845231 10.4103/NRR.NRR-D-23-01756 PMC11438337

[39] Jong Huat T, Camats-Perna J, Newcombe EA, The impact of astrocytic NF-κB on healthy and Alzheimer’s disease brains. Scientific Reports. 2024;14(1):14305. 38906984 10.1038/s41598-024-65248-1 PMC11192733

[40] Li K, Ling H, Wang X, The role of NF-κB signaling pathway in reactive astrocytes among neurodegeneration after methamphetamine exposure by integrated bioinformatics. Prog Neuropsychopharmacol Biol Psychiatry. 2024;129:110909. 38061485 10.1016/j.pnpbp.2023.110909

[41] Hammond SL, Bantle CM, Popichak KA, NF-κB Signaling in Astrocytes Modulates Brain Inflammation and Neuronal Injury Following Sequential Exposure to Manganese and MPTP During Development and Aging. Toxicol Sci. 2020;177(2):506–520. 32692843 10.1093/toxsci/kfaa115 PMC7548288

[42] Aragón-González A, Shaw PJ, Ferraiuolo L. Blood-Brain Barrier Disruption and Its Involvement in Neurodevelopmental and Neurodegenerative Disorders. Int J Mol Sci. 2022;23(23). 10.3390/ijms232315271 PMC9737531 36499600

[43] Manu DR, Slevin M, Barcutean L, Forro T, Boghitoiu T, Balasa R. Astrocyte Involvement in Blood-Brain Barrier Function: A Critical Update Highlighting Novel, Complex, Neurovascular Interactions. Int J Mol Sci. 2023;24(24). 10.3390/ijms242417146 PMC10743219 38138976

[44] Abbott NJ, Patabendige AA, Dolman DE, Yusof SR, Begley DJ. Structure and function of the bloodbrain barrier. Neurobiol Dis. 2010;37(1):13–25. 19664713 10.1016/j.nbd.2009.07.030

[45] Greene C, Campbell M. Tight junction modulation of the blood brain barrier: CNS delivery of small molecules. Tissue Barriers. 2016;4(1):e1138017. 27141420 10.1080/21688370.2015.1138017 PMC4836485

[46] Kim H, Leng K, Park J, Reactive astrocytes transduce inflammation in a blood-brain barrier model through a TNF-STAT3 signaling axis and secretion of alpha 1-antichymotrypsin. Nature Communications. 2022;13(1):6581. 10.1038/s41467-022-34412-4 PMC9630454 36323693

[47] Kushwaha R, Li Y, Makarava N, Reactive astrocytes associated with prion disease impair the blood brain barrier. bioRxiv. 2023. 10.1016/j.nbd.2023.106264 PMC10494928 37597815

[48] Pan R, Liu W, Liu KJ. MMP-2/9-cleaved occludin promotes endothelia cell death in ischemic stroke. Brain Hemorrhages. 2021;2(2):63–70.

[49] Verkhratsky A, Butt A, Li B, Astrocytes in human central nervous system diseases: a frontier for new therapies. Signal Transduction and Targeted Therapy. 2023;8(1):396. 37828019 10.1038/s41392-023-01628-9 PMC10570367

[50] Kim KA, Kim D, Kim JH, Autophagy-mediated occludin degradation contributes to blood-brain barrier disruption during ischemia in bEnd.3 brain endothelial cells and rat ischemic stroke models. Fluids Barriers CNS. 2020;17(1):21. 32169114 10.1186/s12987-020-00182-8 PMC7071658

[51] Versele R, Sevin E, Gosselet F, Fenart L, Candela P. TNF-α and IL-1β Modulate Blood-Brain Barrier Permeability and Decrease Amyloid-β Peptide Efflux in a Human Blood-Brain Barrier Model. Int J Mol Sci. 2022;23(18). 10.3390/ijms231810235 PMC9499506 36142143

[52] Wang Y, Jin S, Sonobe Y, Interleukin-1β induces blood-brain barrier disruption by downregulating Sonic hedgehog in astrocytes. PLoS One. 2014;9(10):e110024. 25313834 10.1371/journal.pone.0110024 PMC4196962

[53] Yang J, Ran M, Li H, New insight into neurological degeneration: Inflammatory cytokines and blood-brain barrier. Front Mol Neurosci. 2022;15:1013933. 36353359 10.3389/fnmol.2022.1013933 PMC9637688

[54] Jung HK, Ryu HJ, Kim MJ, Interleukin-18 attenuates disruption of brain-blood barrier induced by status epilepticus within the rat piriform cortex in interferon-γ independent pathway. Brain Res. 2012;1447:126–134. 22338606 10.1016/j.brainres.2012.01.057

[55] Zhang Y, Li H, Li Z, Recent advances on the role of pro-inflammatory cytokine interleukin-18 in post-stroke depression. Brain Behavior and Immunity Integrative. 2024;5:100037.

[56] Cao K, Liao X, Lu J, IL-33/ST2 plays a critical role in endothelial cell activation and microglia-mediated neuroinflammation modulation. Journal of Neuroinflammation. 2018;15(1):136. 29728120 10.1186/s12974-018-1169-6 PMC5935936

[57] Kossmann T, Stahel PF, Lenzlinger PM, Interleukin-8 released into the cerebrospinal fluid after brain injury is associated with blood-brain barrier dysfunction and nerve growth factor production. J Cereb Blood Flow Metab. 1997;17(3):280–289. 9119901 10.1097/00004647-199703000-00005

[58] Chaitanya GV, Cromer WE, Wells SR, Gliovascular and cytokine interactions modulate brain endothelial barrier in vitro. Journal of Neuroinflammation. 2011;8(1):162. 22112345 10.1186/1742-2094-8-162 PMC3248576

[59] Filipello F, Goldsbury C, You SF, Locca A, Karch CM, Piccio L. Soluble TREM2: Innocent bystander or active player in neurological diseases? Neurobiology of Disease. 2022;165:105630. 35041990 10.1016/j.nbd.2022.105630 PMC10108835

[60] Zhong L, Chen XF. The Emerging Roles and Therapeutic Potential of Soluble TREM2 in Alzheimer’s Disease. Front Aging Neurosci. 2019;11:328. 32038221 10.3389/fnagi.2019.00328 PMC6988790

[61] Hein TM, Nespoli E, Hakani M, NF-κB activation in astrocytes impairs wound healing after traumatic brain injury in male mice. Nature Communications. 2026;17(1):2323. 10.1038/s41467-026-70304-7 PMC12976058 41792174

[62] Ben Haim L, Ceyzériat K, Carrillo-de Sauvage MA, The JAK/STAT3 pathway is a common inducer of astrocyte reactivity in Alzheimer’s and Huntington’s diseases. J Neurosci. 2015;35(6):2817–2829. 25673868 10.1523/JNEUROSCI.3516-14.2015 PMC6605603

[63] Renault-Mihara F, Okano H. STAT3-regulated RhoA drives reactive astrocyte dynamics. Cell Cycle. 2017;16(21):1995–1996. 28933592 10.1080/15384101.2017.1377032 PMC5731407

[64] Bonomi CG, Assogna M, Di Donna MG, Cerebrospinal Fluid sTREM-2, GFAP, and β-S100 in Symptomatic Sporadic Alzheimer’s Disease: Microglial, Astrocytic, and APOE Contributions Along the Alzheimer’s Disease Continuum. J Alzheimers Dis. 2023;92(4):1385–1397. 36911936 10.3233/JAD-221010

[65] Azzolini F, Gilio L, Pavone L, Neuroinflammation Is Associated with GFAP and sTREM2 Levels in Multiple Sclerosis. Biomolecules. 2022;12(2). 10.3390/biom12020222 PMC8961656 35204724

[66] Jayakumar AR, Curtis KM, Panickar KS, Shamaladevi N, Norenberg MD. Decreased STAT3 Phosphorylation Mediates Cell Swelling in Ammonia-Treated Astrocyte Cultures. Biology (Basel). 2016;5(4). 10.3390/biology5040048 PMC5192428 27918421

[67] Ahmed A, Wong M, Santamaria A, JAK2/STAT3 Signaling Pathway Modulates Acute Methylmercury Toxicity in the Mouse Astrocyte C8-D1A Cell Line. Neurochem Res. 2025;50(4):265. 40802001 10.1007/s11064-025-04507-7 PMC12350482

[68] Ghosh S, Liu H, Yazdankhah M, βA1-crystallin regulates glucose metabolism and mitochondrial function in mouse retinal astrocytes by modulating PTP1B activity. Communications Biology. 2021;4(1):248. 33627831 10.1038/s42003-021-01763-5 PMC7904954

[69] Sugiyama S, Sasaki T, Tanaka H, The tight junction protein occludin modulates blood–brain barrier integrity and neurological function after ischemic stroke in mice. Scientific Reports. 2023;13(1):2892. 36806348 10.1038/s41598-023-29894-1 PMC9938878

[70] Pan R, Yu K, Weatherwax T, Zheng H, Liu W, Liu KJ. Blood Occludin Level as a Potential Biomarker for Early Blood Brain Barrier Damage Following Ischemic Stroke. Scientific Reports. 2017;7(1):40331. 28079139 10.1038/srep40331 PMC5228160

[71] Castro V, Bertrand L, Luethen M, Occludin controls HIV transcription in brain pericytes via regulation of SIRT-1 activation. Faseb j. 2016;30(3):1234–1246. 26601824 10.1096/fj.15-277673 PMC4750406

[72] Torices S, Roberts SA, Park M, Malhotra A, Toborek M. Occludin, caveolin-1, and Alix form a multiprotein complex and regulate HIV-1 infection of brain pericytes. Faseb j. 2020;34(12):16319–16332. 33058236 10.1096/fj.202001562R PMC7686148

[73] Torices S, Daire L, Simon S, Occludin: a gatekeeper of brain Infection by HIV-1. Fluids and Barriers of the CNS. 2023;20(1):73. 37840143 10.1186/s12987-023-00476-7 PMC10577960

[74] Liu X, Su P, Meng S, Role of matrix metalloproteinase-2/9 (MMP2/9) in lead-induced changes in an in vitro blood-brain barrier model. Int J Biol Sci. 2017;13(11):1351–1360. 29209140 10.7150/ijbs.20670 PMC5715519

[75] Liu J, Jin X, Liu KJ, Liu W. Matrix metalloproteinase-2-mediated occludin degradation and caveolin-1-mediated claudin-5 redistribution contribute to blood-brain barrier damage in early ischemic stroke stage. J Neurosci. 2012;32(9):3044–3057. 22378877 10.1523/JNEUROSCI.6409-11.2012 PMC3339570

[76] Kim HR, Park JS, Karabulut H, Yasmin F, Jun CD. Transgelin-2: A Double-Edged Sword in Immunity and Cancer Metastasis. Front Cell Dev Biol. 2021;9:606149. 33898417 10.3389/fcell.2021.606149 PMC8060441

